# Sri Lankan tsunami refugees: a cross sectional study of the relationships between housing conditions and self-reported health

**DOI:** 10.1186/1472-698X-9-16

**Published:** 2009-08-05

**Authors:** Alex Turner, Sameera Pathirana, Amanda Daley, Paramjit S Gill

**Affiliations:** 1 Primary Care Clinical Sciences, University of Birmingham, Birmingham B15 2TT, UK; 2 The Impakt Aid Trust, 150/2, Bauddhaloka Mawatha, Colombo 04, Sri Lanka

## Abstract

**Background:**

On the 26^th ^December 2004 the Asian tsunami devastated the Sri Lankan coastline. More than two years later, over 14,500 families were still living in transitional shelters. This study compares the health of the internally displaced people (IDP), living in transitional camps with those in permanent housing projects provided by government and non-government organisations in Sri Lanka.

**Methods:**

This study was conducted in seven transitional camps and five permanent housing projects in the south west of Sri Lanka. Using an interviewer-led questionnaire, data on the IDPs' self-reported health and housing conditions were collected from 154 participants from transitional camps and 147 participants from permanent housing projects. Simple tabulation with non-parametric tests and logistic regression were used to identify and analyse relationships between housing conditions and the reported prevalence of specific symptoms.

**Results:**

Analysis showed that living conditions were significantly worse in transitional camps than in permanent housing projects for all factors investigated, except 'having a leaking roof'. Transitional camp participants scored significantly lower on self-perceived overall health scores than those living in housing projects. After controlling for gender, age and marital status, living in a transitional camp compared to a housing project was found to be a significant risk factor for the following symptoms; coughs OR: 3.53 (CI: 2.11–5.89), stomach ache 4.82 (2.19–10.82), headache 5.20 (3.09–8.76), general aches and pains 6.44 (3.67–11.33) and feeling generally unwell 2.28 (2.51–7.29). Within transitional camp data, the only condition shown to be a significant risk factor for any symptom was household population density, which increased the risk of stomach aches 1.40 (1.09–1.79) and headaches 1.33 (1.01–1.77).

**Conclusion:**

Internally displaced people living in transitional camps are a vulnerable population and specific interventions need to be targeted at this population to address the health inequalities that they report to be experiencing. Further studies need to be conducted to establish which aspects of their housing environment predispose them to poorer health.

## Background

On the 26^th ^December 2004 an earthquake off the coast of Sumatra triggered a tsunami that caused destruction on an unprecedented scale, affecting countries in two continents and an estimated five million people [1]. In Sri Lanka official records note that over 31,000 died, 4,000 were reported missing and over 1/2 million people were displaced consequently [2]. Many internally displaced people (IDP) were housed in the 326 refugee camps scattered along the coastline [3].

IDP in a camp setting often have crude mortality rates (an indicator of health) at least twice that of their pre-displaced baseline level [4]. Most morbidity and mortality is caused by largely preventable and treatable diseases; diarrhoeal diseases, respiratory tract infections, measles and malaria account for 51–95% of all deaths [4-6]. However, much of the existing refugee health research has focused on African war and famine refugees, the tsunami refugee situation was an entirely different setting.

Two years on, many of the transitional camps constructed to house the IDP had been decommissioned, with the occupants living in permanent housing projects provided by the Sri Lankan government or non-government organizations (NGOs). However, over 14,000 families remain in transitional camps [7]. A typical transitional shelter is a small terraced one or two roomed wooden hut with a corrugated iron roof. The huts are poorly ventilated and have no chimney. The camps themselves are extremely crowded and house between 100 and 1,500 people [8,9] with communal water and sanitation facilities. In contrast the purpose built concrete houses in the housing projects have four to six rooms, including a cooking area with a chimney, a private toilet and often piped mains water and a garden.

### Health and housing

The relationship between the standard of housing and health has been recognized for over 150 years [10]. In studies in developed countries, the presence of dampness and mould have been linked to a range of symptoms and illnesses including aches and pains, digestive disorders, and particularly to respiratory tract infections [11-13]. Overcrowding is thought to increase vulnerability to communicable diseases although it has proved difficult to eliminate confounding factors [14,15].85% of Sri Lankans rely on the burning of biomass (wood and coal) for their domestic energy and this process has been linked to a wide range of respiratory diseases [16-18]. This may be exacerbated by the lack of ventilation in the transitional camp housing.

Whilst there is no literature that has focused specifically on Sri Lankan IDP, four studies conducted in other developing countries provide relevant observations of the health effects of the slums of New Delhi [19], Palestinian refugee camps [20,21] and housing in Bangkok [10]. Populations in these studies have comparable living conditions and household densities to the transitional camps. Gender and age were implicated as factors affecting symptom prevalence, both physical and mental [10,19,21]. Both the studies from Palestinian refugee camps found that objective housing conditions, specifically crowding (number of people per room) and dampness, were related to health, particularly respiratory tract infections [20,21] In contrast, Ruback and Pandy found in New Delhi that objective measures of housing conditions related to a participant's mental well-being but not physical health [19]. While in Bangkok, Fuller, Edwards et al found that only self-perceived conditions, including crowding, were related to mental and physical health [10]. However differing study designs make comparisons difficult.

This study describes the conditions in the transitional camps and housing projects, and compares the health of those still living in the camps to that of those who have been relocated to the housing projects. Residents of housing projects were used as a comparison because, pre-tsunami they originate from a similar socio-economic background as those in transitional shelters. The escalating conflict in the north east of Sri Lanka is currently displacing thousands of people, making research on the consequences of living in transitional camps in this region crucial [22].

## Methods

Data for this study was collected in seven transitional camps and five permanent housing projects in the Galle, Panadura and Kalutara districts of southwest Sri Lanka in February and March 2007. Ethical approval for the study was obtained from the South Birmingham Student Ethics Committee in England and the Colombo Medical Faculty Ethical Review Committee in Sri Lanka.

### Study population

Access to the transitional camps was facilitated by Impakt Aid, a Non Government Organisation (NGO) that provides medical clinics to camps in the area. There are no exact figures on how many people are in each transitional camp. The study recruited 20–25 participants from each camp and 30 from each housing project. Each household in a transitional camp or housing project had a number. Random number tables were used to select households, and all participants aged over 18 in the selected household were invited to participate, after written informed consent had been obtained. Using this method no more than three participants were interviewed from any one household. If an eligible individual was not present at the house at the time of visiting two further attempts were made to contact them. No incentives for participation were offered.

### Survey

The survey was conducted using a 74-item pre-piloted structured questionnaire divided into: demographic information; housing and living conditions and current and pre-tsunami health. The health section included a list of symptoms a participant might have had in the last month. These were identified from background research as associated with living conditions [10,11,13] and included; coughs or wheezing, stomach ache, diarrhoea, head ache, general aches and pains and feeling generally unwell. Symptoms were recorded in one of five boxes ranging from 'none of the time', to 'all the time', however for statistical analysis this had to be converted to binary data. Overall health was assessed using a scale ranging from 1 (very poor) to 5 (very good). The questionnaire was translated with the help of a Singhalese-speaking doctor to ensure the questions were given cultural sensitivity and relevance. It was then independently back-translated. The questionnaire was administered by two Sinhalese pre-intern doctors who were monitored to ensure compliance with the study protocol by one of the authors.

### Sample size and statistics

Post hoc power calculations have indicated that the sample size of 303 provides 90% power (p < 0.05) for all the primary outcomes (except diarrhoea). The data were dual entered and inconsistencies corrected. Simple tabulation was used with non-parametric tests to investigate the significance of the differences in living conditions and health between residents of transitional camps and housing projects. Using the Symptoms as dependent variables, unadjusted odds ratios for each explanatory variable were calculated. Forward binary logistic regression was used to calculate adjusted odds ratios for each symptom of living in a transitional camp relative to a housing project. Adjusted odds ratios were checked using backward binary logistic regression.

## Results

### Sample characteristics

The sample consisted of 303 individuals, 60.1% of which were female. The mean age of the sample was 38 years. There were no significant differences in household income or unemployment rates between residents of the transitional camps or housing projects. In both populations the average monthly household income was significantly below the Sri Lankan national average of US$186.45 and unemployment rates were higher than national rates for men and women of 4.9 and 10.3 respectively [23,24]. The housing conditions in the transitional camps were reported to differ considerably from those of the housing projects. Significantly higher frequencies of damp, mould and flooding were reported in the transitional camp homes and the presence of a private toilet and separate kitchen with a chimney in the housing project homes. The study sample characteristics and housing conditions are summarised in Table 1.

**Table 1:** Comparison of sample characteristics and housing condition between transitional camps and permanent housing project.

Demographics		Transitional camp (n = 156)	Housing project (n = 147)	P
Age		37.0 (S.D. 11.8)	38.8 (S.D. 15.1)	0.76

Sex	Male	37% (57)	44% (64)	0.21
		
	Female	63% (99)	56% (83)	

Marital status	Single	5.8% (9)	6.9% (10)	
		
	Married	80.5 (124)	85.5% (124)	0.17
		
	Widowed	11.0% (17)	7.6% (11)	
		
	Divorced	2.6% (4)	-	

Males that smoke tobacco		61.4% (35)	40.1% (26)	0.02

Males that use alcohol Use		37.5% (21)	26.6% (17)	0.20

Household level (means)				

Monthly income		Rs 6921 (S.D. 3830)	Rs 7356 (S.D. 3970)	0.34

Unemployment rates	Male	7% (4)	7.9% (8)	0.87
	
	Female	82.8% (82)	80.7% (67)	0.85

Adults		2.8 (S.D. 1.2)	2.7 (S.D. 1.1)	0.22

Children		1.4 (S.D. 1.0)	1.7 (S.D. 1.3)	0.093

People/room		3.4 (S.D. 1.5)	1.1 (0.4)	<0.001

Housing conditions				

Leaking house		48.7% (76)	53.7% (79)	0.38

Flood when rains	All of the time	5.1% (8)	0.7% (1)	
		
	Some of the time	47.4% (74)	9% (13)	<0.001
		
	None of the time	47.4% (74)	90.3% (131)	

Damp	Very	3.2% (5)	0	
		
	Quite	59.0% (92)	17.2% (25)	<0.001
		
	None	37.8% (59)	59.5% (120)	

Mould	Very	5.1% (8)	0	
		
	Quite	22.4% (35)	13.8% (20)	0.002
		
	None	72.4% (113)	86.2% (125)	

Cooking fuel	Wood	61.3% (92)	81.9% (118)	
		
	Gas	5.3% (8)	1.4% (2)	0.003
		
	Kerosene	31.3% (2)	15.3 (22)	
		
	Combination	1.5% (2)	1.4% (2)	

Drinking water source	Private piped	0	39.5% (58)	
		
	Communal piped	87.8% (136)	36.1% (53)	<0.001
		
	Communal well	0	24.5% (36)	
		
	Bowser	12.3% (19)	0	

Toilet location	Private, inside house	0	67.3% (99)	
		
	Private, outside house	0	32.7% (48)	<0.001
		
	Communal	100% (156)	0	

Number using toilet		222.6 (S.D. 208.2)	4.4 (S.D. 1.8)	<0.001

### Housing, overall health and symptom prevalences

There were no significant differences between any of the seven transitional camps' mean overall health scores or symptom prevalences, the same was true for the five housing project locations. Transitional camp and housing project mean pre-tsunami overall health scores were not significantly different. However residents in transitional camps rated their current health significantly lower than residents in housing projects; p < 0.005 for men and p < 0.001 for women. The prevalence of all reported symptoms excluding diarrhoea was significantly higher in the transitional camps than the housing projects. Feeling generally unwell was the most prevalent symptom and was reported by 64.9% of males and 83.8% of females in the transitional camps compared to 39.1% and 50.6% of males and females in the housing projects. Coughs, stomach aches, headaches and general aches and pains were reported over twice as prevalent in the transitional camps. (Table 2).

**Table 2:** Pre-tsunami and current overall health scores and prevalence of symptoms suffered in the last month.

Symptom/health score						
	Male			Female		
	
	Transitional camp (n = 57)	Housing project (n = 64)	P	Transitional camp (n = 99)	Housing project (n = 83)	P

Overall current health (mean)^a^	2.86 (S.D. 1.27)	3.53 (S.D. 1.10)	0.004	2.83 (S.D. 1.00)	3.65 (S.D. 0.97)	<0.001

Overall pre-tsunami health (mean)^a^	4.12 (S.D. 0.69)	3.95 (S.D. 0.88)	0.24	4.11 (S.D. 0.65)	3.88 (S.D. 0.90)	0.051

Unwell	64.9% (37)	39.1% (25)	0.005	83.8% (83)	50.6% (42)	<0.001

Cough	43.9%(25)	21.9% (14)	0.01	52.5% (52)	20.5% (17)	<0.001

Stomach ache	15.8% (9)	7.8% (5)	0.17	25.3% (25)	3.6% (3)	<0.001

Diarrhoea	5.3% (3)	6.3% (4)	0.82	8.1% (8)	3.6% (3)	0.21

Headache	40.4% (23)	18.8% (12)	<0.01	71.7% (71)	26.5% (22)	<0.001

General aches and pains	50.9% (29)	21.9% (14)	<0.01	61.6% (61)	22.9% (19)	<0.001

^a^The differences between pre-tsunami and current health for each group was significant (p < 0.001).

Table 3 shows adjusted risk factors for each symptom, controlling for age, gender, marital status and income, of living in a transitional camp compared to a housing project. Living in a transitional camp compared to a housing project was found to be a high and significant risk factor for the following symptoms; (O.R. 95% C.I.); coughs 3.53 (2.11–5.89), stomach ache 4.82 (2.19–10.82), headache 5.20 (3.09–8.76), general aches and pains 6.44 (3.67–11.33) and feeling generally unwell 2.28 (2.51–7.29). See Table 3.

**Table 3:** Logistic regression estimates of adjusted risk factors of symptoms comparing transitional camps to housing projects.

Independent variables	Dependant variables					
	Unwell	Cough	Stomach ache	Diarrhoea	Headache	General aches and pains
	
	Exp. (B) (95% CI)	Exp. (B) (95% CI)	Exp. (B) (95% CI)	Exp. (B) (95% CI)	Exp. (B) (95% CI)	Exp. (B) (95% CI)

Age (yrs) (base value 18, unit yrs)	1.03 (1.01–1.05)**	-	-	-	-	1.06 (1.03–1.08)***

Male	1	-	-	-	1	-

Female	2.28 (2.52–7.29)**	-	-	-	2.56 (1.50–4.36)**	-

Single	1	-	-	-	-	-

Married	0.25 (0.08–0.80)*	-	-	-	-	-

Widowed	0.67 (0.11–4,18)	-	-	-	-	-

Divorced	0.13 (0.01–2.03)	-	-	-	-	-

Housing project	1	1	1	-	1	1

Transitional camp	2.28 (2.51–7.29)***	3.53 (2.11–5.89)***	4.82 (2.19–10.82)***	-	5.20 (3.09–8.76)***	6.44 (3.67–11.33)***

*p < 0.05, **p < 0.01, ***p < 0.001

Due to the high correlation between housing conditions and housing type, ie. transitional camp or housing project, the data from the transitional camps had to be analysed separately to determine which conditions of the environment were risk factors for the symptoms [25]. All the independent variables were entered and the significant relationships are shown in Table 4. The only predictive factor was household population density, which was a significant risk factor for stomach ache 1.40 (1.09–1.79) and headaches 1.33 (1.01–1.77). See Table 4

**Table 4:** Logistic regression estimates of the risk factors of each symptom among residents of transitional camps.

Independent variables		Dependant variables					
		Unwell	Cough	Stomach ache	Diarrhoea	Headache	General aches and pains
		
		Exp. (B) (95% CI)	Exp. (B) (95% CI)	Exp. (B) (95% CI)	Exp. (B) (95% CI)	Exp. (B) (95% CI)	Exp. (B) (95% CI)

Age (yrs) (base value 18, unit yrs)		-	-	-	-	-	1.06 (1.03–1.09)***

Male		1	-	-	-	1	-

Female		2.93 (1.33–6.45)**	-	-	-	4.80 (2.09–11.03)***	-

People/room (base value 0.5, units 1 P/r)		-	-	1.40 (1.09–1.79)***	-	1.33 (1.01–1.77)*	-

Mould	None	-	-	-	-	1	-
	
	Some	-	-	-	-	0.70 (0.30–3.89)	-
	
	Lots	-	-	-	-	3.63 (1.30–10.15)*	-

*p < 0.05, **p < 0.01, ***p < 0.001

## Discussion

This study shows the self-reported health of the IDP still living in the transitional camps to be significantly worse in comparison to the IDP living in permanent housing projects. These differences are present despite the limited variation between demographic and socio-economic factors of the two populations.

### Housing conditions and symptom prevalence

Residents of transitional camps were more likely to report experiencing all symptoms investigated, (excluding diarrhoea) than those living in permanent housing. A possible increased prevalence of viral infections in the transitional camps could provide an explanation for the results. The poor housing conditions in the camps such as high household density and communal services could compound transmission of a viral factor. The lack of a chimney in any of households in the camps could account for the increased prevalence of coughs among camp residents [20,21]. It is interesting to note that the symptoms with very high odds ratios (headaches, stomach aches and general aches and pains) are often thought not to be primarily organic in origin [26,27]. Low socio-economic status and slow socio-economic recovery following a natural disaster [28,29], as well as the transitional camps themselves [30], have all been shown to be risk factors for poor psychological health outcomes. Carballo, Heal & Hernandez, De Silva, and Fernando have commented that Sri Lankans often present with somatisation rather than classic western symptoms of psychological distress [31-33]. Thus, it is possible that psychological distress associated with living in a transitional camp could explain a proportion of the high prevalence of reported pain symptoms of the IDP living there. This is supported by the association between environmental stressors and increased psychological distress found by Ruback and Pandey [11]. However further research into the mental health of the IDP is required to support this hypothesis.

For the transitional camp data alone, household density (number of residents per room) was shown to be a risk factor for reporting 'stomach aches' and 'headaches'. The association between household density and 'stomach ache' could indicate a relationship between crowding and enteric illness. However, the prevalence of diarrhoea is low, and in light of the high prevalence of other pain symptoms in the transitional camps, it is possible that crowding increases risk of head and stomach ache via a mechanism of increased stress. This significant effect of objective crowding (household density) on either physical or mental health self-reported outcomes is contrary to the findings of Ruback and Pandey in slums of Delhi and of Fuller, Edwards et al in Bangkok (although both studies found an association between perceived crowding and poorer mental health) [10,11]. In the Palestinian refugee camps a significant relationship between household density and poorer health was found, but neither headache nor stomach ache were investigated [12,13]. The tendency for this population to somatise their psychological distress combined with the high household density could suggest that the association between objective crowding and reported physical symptoms in this study operated via the influence of subjective crowding on mental distress found by Ruback and Pandey and Fuller, Edwards et al [10,11,31,32].

Individuals reporting that their homes contained lots of mould were shown to have a significant risk of reporting 'headaches'. However, previous studies investigating the health effects of domestic mould have not shown this relationship [15,16], and due to the low number reporting 'lots' of mould mean the reliability of this finding is low. Other than population density, the results did not uncover which housing conditions of the transitional camps predisposed an individual to suffer from any of the symptoms. It is likely that this is due to the homogeneity of the conditions within the camps. Diarrhoea prevalence was low in both groups which could be due to the adult age group of the sample.

### Age and gender

In line with previous studies, increasing age was found to be a significant independent risk factor affecting reports of 'general aches and pains' and 'feeling unwell' [11]. Women were found to be more at risk of reporting 'feeling generally unwell' and 'headaches' (the ratio of 2.7 found in this study being within the range of 1.1 and 3.1 found in other studies [26,34]. It has been shown that women are more likely to experience poor mental health after a disaster [28], which may explain some of the increased self-perceived morbidity risk for women in this study.

### Methodological considerations

Although the most appropriate method for this study, the cross sectional design does not allow for causal inference between housing and health. Attempts were made to minimise selection bias by including all locations where access could be provided, however this was not possible in all areas so generalisability may be limited. Due to the nature of patient-doctor interaction and health care utilisation in this population it was not possible to obtain a reliable disease specific diagnosis for existing medical problems from the participants for this study. Although the reliability of subjective measures of health has often been debated, in epidemiological research self-perceived health has been shown to be a valid measure of morbidity and may even be superior to physician-assessed morbidity in a prognostic sense [35,36]. The survey may have encouraged the over reporting of specific symptoms, however this should have been the same for both populations.

Recall bias was minimised by using a recall period of one month which is acceptable in health interview surveys in developing countries [37]. Due to the inherent recall bias in questioning pre-tsunami health, it can be concluded that participants feel their health has deteriorated, but it cannot be assumed that the difference in pre and current health scores shows an actual difference in health. It is also possible that the IDP in the transitional camps were more likely to exaggerate their ill health and poor living conditions, while those living in housing projects were more inclined to highlight the positive aspects of their home and health.

This is the first study to investigate the housing conditions and health (self-reported or otherwise) of this population. It is also the first to compare the health of IDP living in different types of housing provided after a disaster. The study had a 100% response rate among contacted participants and the data demonstrated internal consistency with expected patterns in terms of sex and age. Whilst similar studies have looked at one specific condition or a single overall health outcome, this study has examined a broader range of self-reported symptoms and has shown the risk factors of living in transitional camps to vary considerably with the symptom.

## Conclusion

Two years after the tsunami, people living in the transitional shelters are still disadvantaged and vulnerable. This study demonstrates that the IDP in the transitional camps perceive their health to be markedly poorer than those that have been relocated. Levels of housing conditions deemed unhealthy are considerably higher in transitional camps than housing projects, and living in a transitional camp seems to be a high risk factor for a number of symptoms. The very high odds ratios of reported pain symptoms, the post-disaster situation and this particular populations' tendency to somatise, suggests that a proportion of the high prevalence rate is possibly due to psychological distress. On the basis of these findings greater emphasis needs to be put on moving people into housing projects as soon as possible to ensure that their health and well being is not further compromised. Further investigation also needs to be conducted into the prevalence of specific conditions, chronic diseases and mental health in the transitional camps. A larger study using objective measures would provide resource allocators with the information required to most effectively provide interventions to improve the health of this population.

## Competing interests

The authors declare that they have no competing interests.

## Authors' contributions

AT conceived the study, participated in the design, implementation, analysis and helped draft the manuscript. SP participated in the study design and implementation. PG and AD participated in the design, interpreted the data and helped draft the manuscript. All authors have read and approved the final manuscript.

## Pre-publication history

The pre-publication history for this paper can be accessed here:

http://www.biomedcentral.com/1472-698X/9/16/prepub
